# Disulfide bond engineering of AppA phytase for increased thermostability requires co-expression of protein disulfide isomerase in *Pichia pastoris*

**DOI:** 10.1186/s13068-021-01936-8

**Published:** 2021-03-31

**Authors:** Laura Navone, Thomas Vogl, Pawarisa Luangthongkam, Jo-Anne Blinco, Carlos H. Luna-Flores, Xiaojing Chen, Juhani von Hellens, Stephen Mahler, Robert Speight

**Affiliations:** 1 Faculty of Science, Queensland University of Technology, Brisbane, QLD Australia; 2 ARC Centre of Excellence in Synthetic Biology, Queensland University of Technology, Brisbane, QLD Australia; 3 Department of Computer Science and Applied Mathematics, Weizmann Institute of Science, Rehovot, Israel; 4 Bioproton Pty Ltd, Brisbane, QLD Australia; 5 ARC Training Centre for Biopharmaceutical Innovation, The University of Queensland, Brisbane, QLD Australia

**Keywords:** Phytase, Thermostability, Disulfide bond, Chaperone, Folding

## Abstract

**Background:**

Phytases are widely used commercially as dietary supplements for swine and poultry to increase the digestibility of phytic acid. Enzyme development has focused on increasing thermostability to withstand the high temperatures during industrial steam pelleting. Increasing thermostability often reduces activity at gut temperatures and there remains a demand for improved phyases for a growing market.

**Results:**

In this work, we present a thermostable variant of the *E. coli* AppA phytase, ApV1, that contains an extra non-consecutive disulfide bond. Detailed biochemical characterisation of ApV1 showed similar activity to the wild type, with no statistical differences in *k*_cat_ and *K*_M_ for phytic acid or in the pH and temperature activity optima. Yet, it retained approximately 50% activity after incubations for 20 min at 65, 75 and 85 °C compared to almost full inactivation of the wild-type enzyme. Production of ApV1 in *Pichia pastoris* (*Komagataella phaffi*) was much lower than the wild-type enzyme due to the presence of the extra non-consecutive disulfide bond. Production bottlenecks were explored using bidirectional promoters for co-expression of folding chaperones. Co-expression of protein disulfide bond isomerase (Pdi) increased production of ApV1 by ~ 12-fold compared to expression without this folding catalyst and restored yields to similar levels seen with the wild-type enzyme*.*

**Conclusions:**

Overall, the results show that protein engineering for enhanced enzymatic properties like thermostability may result in folding complexity and decreased production in microbial systems. Hence parallel development of improved production strains is imperative to achieve the desirable levels of recombinant protein for industrial processes*.*

**Supplementary Information:**

The online version contains supplementary material available at 10.1186/s13068-021-01936-8.

## Background

Phytases (myo-inositol hexakisphosphate phosphohydrolases) catalyse the stepwise removal of phosphate groups from phytate, the main form of phosphorous storage in grains. Phytases are commonly added to livestock feed, reducing the addition of inorganic phosphate to diets and decreasing the anti-nutritional effects of phytate [8, 9]. Phytases in the livestock feed market are derived from the fungal or bacterial origin, namely *Aspergillus niger*, *Escherichia coli*, *Peniophora lycii*, *Citrobacter braakii* and *Butioauxella* spp., and some have been engineered to improve enzymatic properties [17, 19, 36, 39, 46, 50, 62]. Among commercially available phytases, *E. coli* AppA phytase shows high catalytic efficiency and has been extensively improved through engineering [31]. The three main desirable characteristics for phytase enzyme supplements are high specific activity in the gut, high stability during storage and feed pellet steam extrusion, and high levels of production in industrial microbial fermentations [13, 50]. Enzyme stability is a key factor. Activity can be lost through thermal denaturation during feed pellet production where the enzyme may typically be exposed to temperatures approaching ~ 90 °C for up to 90 s during storage and transportation, and through degradation by proteases and the acidic conditions encountered in the gastrointestinal tract.

Increased stability of enzymes can be achieved through protein engineering, and there are several examples of mutagenesis for phytases [17, 19, 29, 36, 39, 46, 49, 53, 54, 62, 65, 66]. In a prominent instance, Garrett et al. designed an enzyme that was mutated at nine amino acid positions to achieve increased thermostability following a random mutagenesis approach [19, 51]. The enzyme is termed Phy9X, also referred to as NOV9X in the patent literature [30] and Quantum phytase in registration documents [14]. The melting temperature (Tm) for Phy9X was 12 °C above the wild-type AppA phytase. Increasing the *T*_m_ often leads to an upward shift in optimal temperature and therefore reduced activity at mesophilic temperatures, potentially affecting enzymatic activity at the normal body temperature of animals.

Increasing thermal stability through the incorporation of additional disulfide bonds is a common strategy in protein engineering and the basis of this effect has been examined using a range of phytases [44, 61]. For example, the native *E. coli* AppA phytase has four disulfide bonds and including three additional bonds was shown to increase the melting temperature by 8.5 °C and the optimal temperature for activity to 75 °C from 53 °C [11]. In this work, cysteine residues were incorporated at positions G74C/A121C, T163C/V222C and D53C/L199C to form three non-consecutive disulfide bonds. Increasing the number of disulfide bonds in a protein structure for improved thermostability can lead to protein folding and secretion difficulties in the production microorganism. Disulfide bonds formed between incorrect cysteine partners is detrimental to correct enzyme folding and therefore activity. Folding chaperones and other proteins, for example, protein disulfide isomerases, can be co-expressed with the recombinant enzyme to improve expression and disulfide bond formation [28, 35, 45, 55].

In this work, we present a new thermostable variant of *E. coli* AppA phytase, ApV1. The variant has an additional non-consecutive disulfide bond compared to the wild-type AppA phytase, leading to a total of five disulfide bonds. The presence of the extra disulfide bond in ApV1 increased residual activity after heat treatment despite presenting the same temperature profile and kinetic properties as the wild-type AppA phytase.

While the additional disulfide bond in ApV1 improved thermal stability it markedly decreased expression in the host *P. pastoris* to levels not suitable for economic manufacturing at an industrial scale. We use a synergistic approach to engineer *P. pastoris* strains and optimise the production of the thermostable ApV1 phytase. Our previous investigations show that the production of AppA phytase in *P. pastoris* was greatly improved using bidirectional promoters (BDPs) [38, 56]. Here, BDPs were used for co-expression of chaperones to increase production of ApV1 phytase. The highest production yields were obtained when co-expressing *PDI* suggesting that the isomerase is essential to circumvent folding complexity and enable the correct formation of the additional disulfide bond in ApV1. Modifications of the native amino acid sequence of proteins to improve enzymatic properties may at the same time lead to undesirable properties such as decreased production. Our results show that parallel approaches of protein and strain engineering interdependently complement each other to deliver improved enzymes in acceptable yields.

## Results

### Thermostability studies of ApV1 phytase

**Fig. 1:**
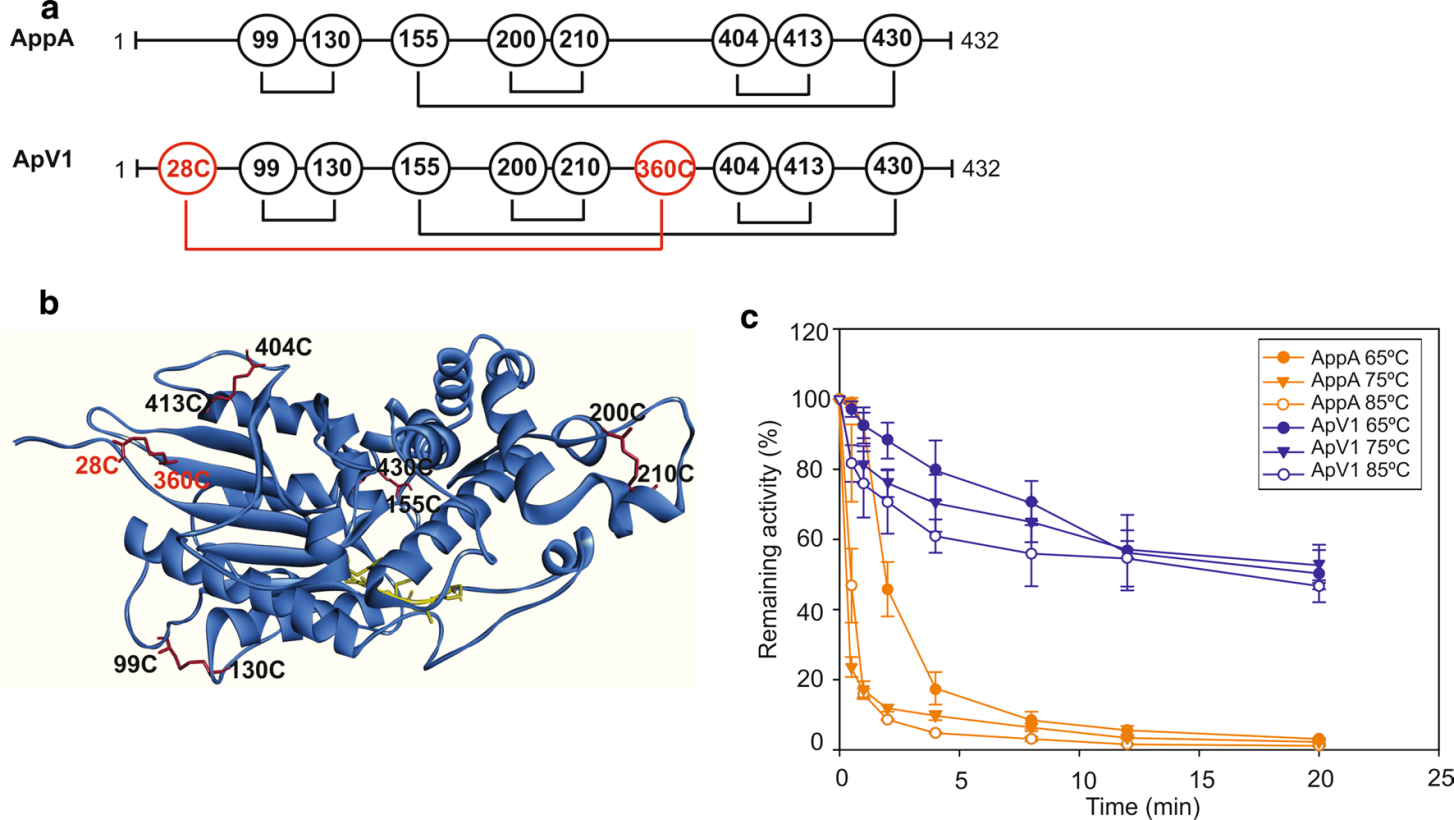
ApV1 shows improved thermostability at 65, 75 or 85 °C. Diagram of AppA phytase amino acid sequence indicating cysteine residues forming disulfide bonds and residue mutations in ApV1 (L28C, W360C) (**a**). ApV1 structure indicating disulfide bonds (in red) and active site residues (in yellow) (**b**). ApV1 and AppA thermostability curves at 65, 75 or 85 °C (**c**). Phytase activity was determined by the p-NPP assay after incubation at 65, 75 or 85 °C. Remaining activity was calculated as a percentage of the observed phytase activity with no incubation. All assays were performed at 37 °C. Data are represented as mean values ± standard deviation (*n* = 3)

**Fig. 2:**
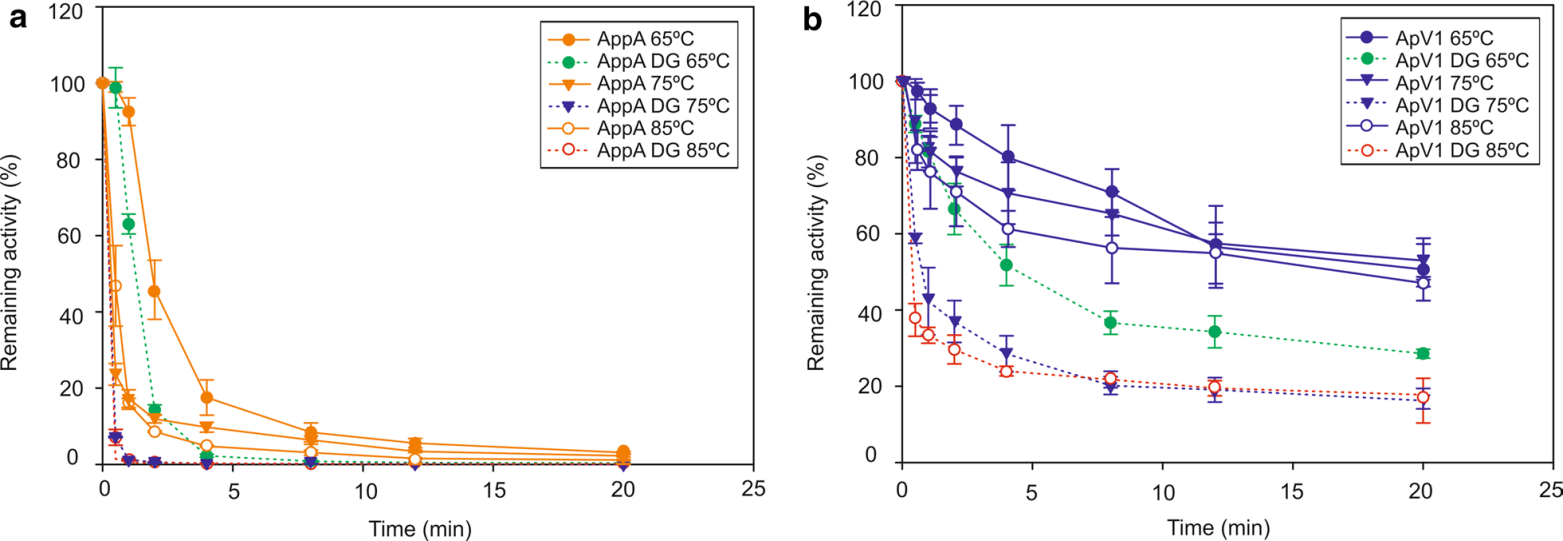
ApV1 phytase retains the thermostability improvement after deglycosylation although glycosylation contributes to overall stability. Remaining activity of glycosylated and deglycosylated AppA (**a**) and ApV1 (**b**) phytases. Phytase activity was determined by the p-NPP assay after incubation at 65, 75 or 85 °C. Remaining activity was calculated as a percentage of phytase activity without high-temperature treatment. Deglycosylated forms are indicated as “DG”. Data are represented as mean values ± standard deviation (*n* = 3)

**Fig. 3:**
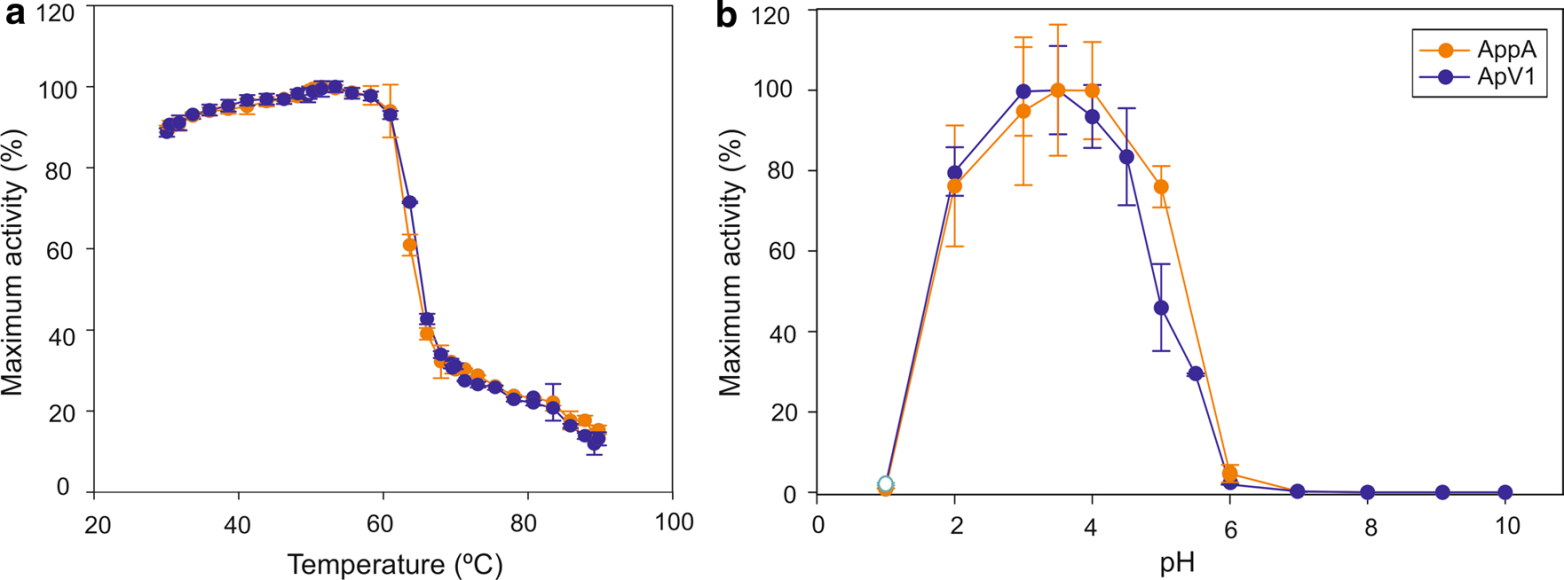
ApV1 displays indistinguishable optimal temperature and pH activities to AppA phytase. Optimal temperature (**a**) and pH (**b**) curves for ApV1 and AppA phytases. Phytase activity was determined by the p-NPP assay at 37 °C. Percentage of maximum activity was calculated as a percentage of phytase activity at the optimal temperature or pH. Data are represented as mean values ± standard deviation (*n* = 3)

ApV1 thermostable variant was engineered using a rational design based on the available structure of the *E. coli* AppA phytase [32]. The amino acids L28 and W360 in the phytase wild-type sequence were both mutated to cysteines (L28C, W360C) to generate ApV1 with the aim of forming an additional disulfide bond to enhance thermal stability (Fig. 1a, b). Detailed biochemical characterisation was then conducted to study ApV1 properties (Figs. 1b, 2, 3). ApV1 and the wild-type AppA phytase were expressed in *P. pastoris* and His-tag purified for biochemical characterisation. Thermal analysis showed that the incorporation of the extra disulfide bond increased thermostability of the ApV1 phytase compared to AppA phytase during incubation at 65, 75 and 85 °C (Fig. 1c). ApV1 phytase was relatively stable with 50 ± 8% remaining activity after 20 min at 65 °C, 53 ± 4% after 20 min at 75 °C and 47 ± 1% after 20 min at 85 °C. AppA phytase was less stable and showed 3 ± 1%, 1 ± 1% and 2 ± 1% remaining activity after 20 min at 65, 75 and 85 °C, respectively (Fig. 1c).

Disulfide bonds as well as glycosylation patterns contribute to the thermal stability of enzymes [16, 24, 47, 48, 58]. *P. pastoris* adds glycan chains to polypeptides during protein folding [21, 24, 62] and the observed thermal stability profiles of AppA and ApV1 phytases is most likely a contribution of both factors. To study the intrinsic thermal stability of the protein structure without the influence of glycosylation, ApV1 and AppA phytases were deglycosylated using endoglycosidase H. Figure 2 shows a comparison of thermostability curves for the glycosylated and deglycosylated forms of ApV1 and AppA phytases at 65, 75 and 85 °C. Thermal stability of deglycosylated wild-type AppA quickly decreases at all temperatures becoming inactivated after few minutes (Fig. 2a). While the thermal stability of the deglycosylated form of ApV1 phytase decreases at all temperatures compared to the glycosylated form, deglycosylated ApV1 phytase retains 29 ± 1% activity after 20 min incubation at 65 °C, 16 ± 6% at 75 °C and 18 ± 3% at 85 °C (Fig. 2b). ApV1 activity drops to ~ 0% after 4 h incubation at 65, 75 or 85 °C (data not shown).

### Biochemical characterisation of AppA and ApV1 phytases

Specific activity and kinetic parameters of purified ApV1 and AppA phytases were determined using p-NPP and phytic acid as substrates. No significant difference was observed between ApV1 and AppA phytase for any of the kinetic parameters studied (Table 1). ApV1 phytase maintained catalytic activity and *K*_M_ for the different substrates whilst showing increased thermostability, suggesting that the stabilisation mechanism was independent of the catalytic activity of the enzyme.

**Table 1:** Kinetic parameters for ApV1 thermostable phytase compared to AppA phytase at 37 °C

	Specific activity (10^3^ U/g of protein)*	*K*_M_ (mM)*	*k*_cat_ (min^−1^)*	*k*_cat_/*K*_M_ (min^−1^ M^−1^)*
Phytic acid assay				
AppA phytase	1150 ± 138	0.83 ± 0.04	3.91 × 10^5^ ± 0.51 × 10^5^	3.47 × 10^8^ ± 0.34 × 10^8^
ApV1 phytase	1273 ± 238	0.82 ± 0.04	2.65 × 10^5^ ± 0.37 × 10^5^	3.23 × 10^8^ ± 0.28 × 10^8^
p-NPP assay				
AppA phytase	1075 ± 40	10.63 ± 1.94	2.02 × 10^5^ ± 0.64 × 10^5^	1.57 × 10^7^ ± 0.22 × 10^7^
ApV1 phytase	987 ± 42	11.34 ± 0.48	2.45 × 10^5^ ± 0.13 × 10^5^	1.72 × 10^7^ ± 0.86 × 10^7^

*No significant statistical difference between phytases for phytic acid or p-NPP assays (*p* ≤ 0.05). Values correspond to biological triplicates of his-tag purified AppA and ApV1 phytases

Optimal temperature and pH activity profiles calculated relative to the maximum for ApV1 and AppA phytases were very similar (Fig. 3). The optimal temperature was between 50 and 55 °C for the ApV1 and AppA phytases and pH curves showed similar trends for both phytases. ApV1 shows 94% relative maximum activity at 37 °C, showing no difference with AppA relative activity at the same temperature. Both AppA and ApV1 enzymes appear to denature at the same temperature as shown in Fig. 3a, which leads us to hypothesise that the stability shown by ApV1 (Figs. 1 and 2) is most likely due to increased refolding to its active form promoted by the extra 28C–360C bond.

Optimal pH was 4.00 to 4.50 for ApV1 phytase and AppA phytases and both phytases maintained 100% activity after 1 h incubation at 37 °C at pH 2, 3, 4 and 5 (Table 2). Pepsin (a common digestive endopeptidase) resistance was also tested at a 1:1 pepsin/phytase ratio and both enzymes were highly stable, with ApV1 phytase showing 101 ± 2% remaining activity while AppA phytase showed 99 ± 5% remaining activity after 4 h incubations.

**Table 2:** pH stability (%) after 1 h incubation at 37 °C

	pH 2	pH 3	pH 4	pH 5
AppA phytase	98 ± 1	98 ± 2	94 ± 4	105 ± 4
ApV1 phytase	101 ± 4	98 ± 1	94 ± 8	107 ± 8

### Transcriptional optimisation of ApV1 production and the effect of co-expression of *HAC1*

**Fig. 4:**
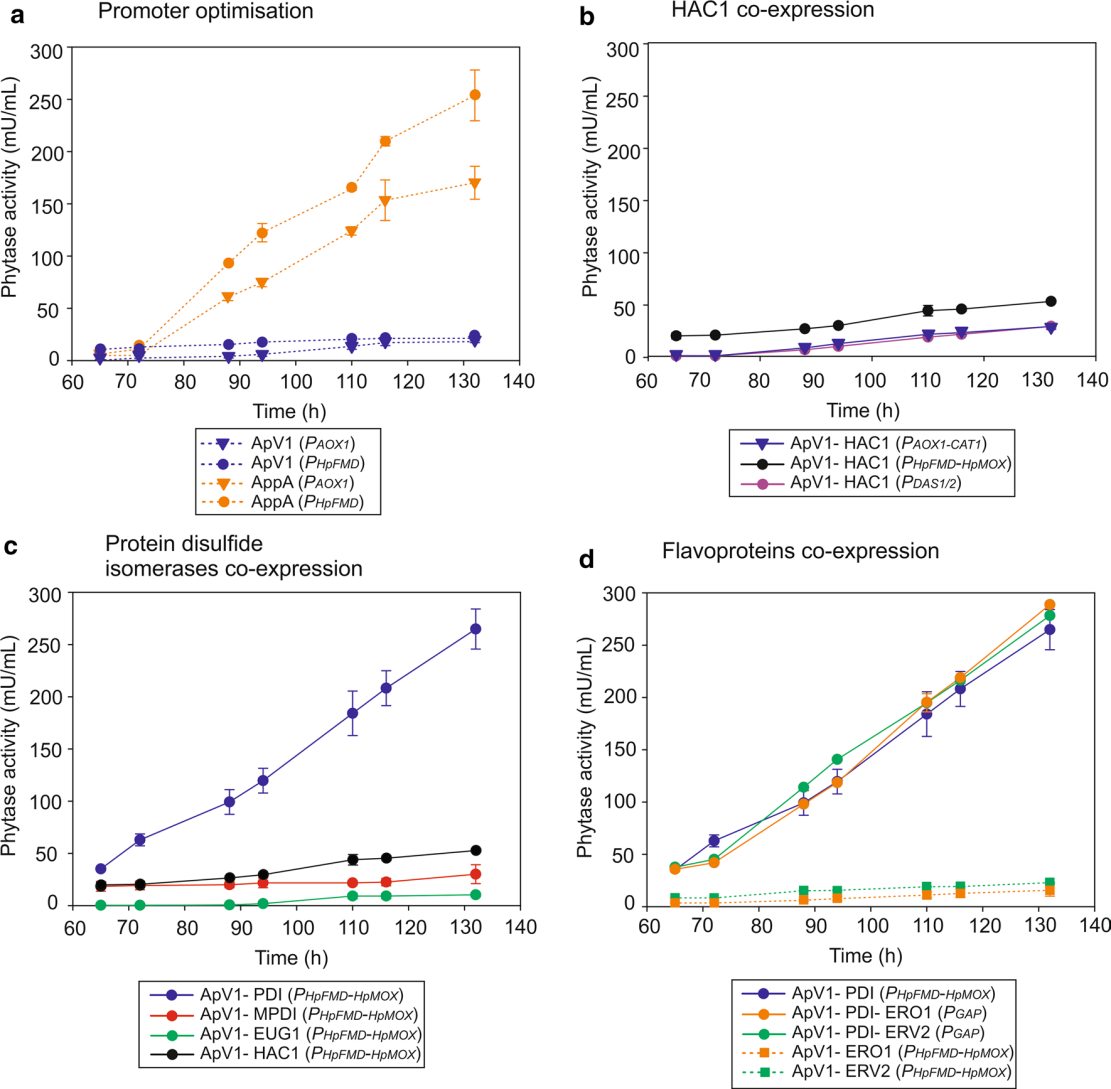
*ApV1* expression from *P*_*HpFMD*_ exceeds expression from *P*_*AOX1*_ and *PDI* co-expression with BDP *P*_*HpFMD-HpMOX*_ shows the highest ApV1 yields. *ApV1* expression from MDPs *P*_*AOX1*_ and *P*_*HpFMD*_ with AppA (*P*_*AOX1*_) with phytase activity (mU/mL) shown on the secondary axis (**a**). *ApV1* co-expression with HAC1 from the BDPs *P*_*AOX1-CAT1*_, *P*_*DAS1/2*_ and *P*_*HpFMD-HpMOX*_ (**b**). *ApV1* co-expression with *PDI*, *MPDI* and *EUG1* under the BDP *P*_*HpFMD-HpMOX*_ (**c**). *ApV1* co-expression with or without *PDI* and flavoproteins *ERO1* and *ERV2* (**d**). Phytase expression was determined by the p-NPP assay. AppA (*P*_*HpFMD*_) is included for comparison. All strains were induced with methanol at 65 h and consecutive methanol additions until 132 h. Data are represented as mean values ± standard deviation (*n* = 3)

The presence of the additional non-consecutive disulfide bond in ApV1 greatly impaired the production of the thermostable phytase compared to AppA (Fig. 4). To improve the expression of ApV1 we followed a similar strategy to the optimisation of AppA production in *P. pastoris* previously reported [38]. The methanol inducible *P*_*AOX1*_ from *P. pastoris* and *P*_*HpFMD*_ from *H. polymorpha* [56] were tested for the expression of ApV1 phytase (Fig. 4a). The ApV1 (*P*_*HpFMD*_) strain showed a 1.18 ± 0.03-fold increase in phytase production compared to the AppA (*P*_*AOX1*_) strain (Table 3). No difference in growth rate was observed between the strains (data not shown). The result followed the same trend reported for AppA phytase production where *P*_*HpFMD*_ surpasses *P*_*AOX1*_ production [38]. Figure 4a shows a comparison between AppA and ApV1 phytases expression with *P*_*HpFMD*_. Expression of AppA is 12-fold greater than the ApV1 thermostable variant, showing the detrimental effect resulting from the extra non-consecutive disulfide bond (Table 3).

**Table 3:** Fold change in ApV1 phytase activity at 132 h relative to ApV1 (*P*_*AOX1*_), ApV1 (P_*HpFMD*_) or ApV1-PDI (P_*HpFMD-HpMOX*_)

Strain	Fold change relative to ApV1 (*P*_*AOX1*_)	Fold change relative to ApV1 (*P*_*HpFMD*_)	Fold change relative to ApV1-PDI (*P*_*HpFMD-HpMOX*_)
AppA (P_*HpFMD*_)	14.01 ± 0.06*	11.91 ± 0.05**	0.96 ± 0.01
ApV1 (P_*AOX1*_)	–	0.85 ± 0.04**	0.07 ± 0.01***
ApV1 (P_*HpFMD*_)	1.18 ± 0.03*	–	0.08 ± 0.01***
ApV1-HAC1 (P_*HpFMD-MOX*_)	2.90 ± 0.05*	2.46 ± 0.05**	0.20 ± 0.01***
ApV1-HAC1 (P_*AOX1- CAT1*_)	1.57 ± 0.15*	1.34 ± 0.13	0.11 ± 0.01***
ApV1-HAC1 (P_*DAS1/2*_)	1.58 ± 0.05*	1.35 ± 0.01	0.11 ± 0.01***
ApV1-PDI (P_*HpFMD-MOX*_)	14.63 ± 1.00*	12.44 ± 0.90**	–
APV1-MPDI (P_*HpFMD-MOX*_)	1.66 ± 0.50*	1.41 ± 0.10**	0.11 ± 0.03***
APV1-EUG1 (P_*HpFMD-MOX*_)	0.57 ± 0.11*	0.48 ± 0.10**	0.04 ± 0.01***
APV1-ERO1 (P_*HpFMD-MOX*_)	0.74 ± 0.14*	0.87 ± 0.17	0.06 ± 0.03***
APV1-ERV2 (P_*HpFMD-MOX*_)	1.27 ± 0.01*	1.08 ± 0.01	0.09 ± 0.01***
APV1-PDI-ERO1 (P_*GAP*_)	15.95 ± 0.05*	13.56 ± 0.05**	1.09 ± 0.03
APV1-PDI-ERV2 (P_*GAP*_)	15.37 ± 0.03*	13.07 ± 0.02**	1.05 ± 0.05
ApV1-PDI *ots1* (P_*HpFMD-MOX*_)	5.82 ± 0.82*	4.95 ± 0.07**	0.40 ± 0.06***
ApV1-PDI *suc2* (P_*HpFMD-MOX*_)	2.06 ± 0.01*	2.21 ± 0.05**	0.18 ± 0.01***
ApV1-PDI *pho1* (P_*HpFMD-MOX*_)	1.73 ± 0.07*	1.47 ± 0.06**	0.12 ± 0.01***

^a^All strains had a single copy of the integrated cassette (CNV = 1) as determined by ddPCR

^*^Significantly different from ApV1 (*P*_*AOX1*_) (*p* value ˂ 0.05)

^**^Significantly different from ApV1 (*P*_*HpFMD*_) (*p* value ˂ 0.05)

^***^Significantly different from ApV1-PDI (*P*_*HpFMD-HpMOX*_) (*p* value ˂ 0.05)

To further optimise expression, *ApV1* phytase was co-expressed with *HAC1*, the master regulator of the unfolded protein response (UPR), under the methanol inducible BDPs *P*_*AOX1-CAT1*_, *P*_*HpFMD-HpMOX*_ and *P*_*DAS1/2*_ (Fig. 4b) [57]. BDPs *P*_*HpFMD-HpMOX*_ and *P*_*AOX1-CAT1*_ are strong methanol inducible promoters on both sides showing mixed regulation that corresponds to each promoter’s inherent expression levels [57]. The BDP *P*_*DAS1/2*_ is naturally occurring in *P. pastoris* and is also methanol inducible but shows the same equally strong expression of both sides [57]. Temporal regulation of expression can be beneficial when it is necessary to express one gene before the other, as in the case presented here for co-expression of folding chaperones with phytases. Furthermore, optimal production of both proteins can be gene pair specific and so it is important to screen for the optimal promoter combination [57]. ApV1-HAC1 (*P*_*HpFMD-HpMOX*_) strain showed a 2.90 ± 0.05-fold increase and 2.46 ± 0.05-fold increase after 132 h compared to ApV1 (*P*_*AOX1*_) and ApV1 (*P*_*HpFMD*_) strains, respectively (Table 3). ApV1-HAC1 (*P*_*AOX1-CAT1*_) and ApV1-HAC1 (*P*_*DAS1/2*_) showed 1.57 ± 0.15 and 1.58 ± 0.05-fold increase in phytase production after 132 h compared to ApV1 (*P*_*AOX1*_), respectively.

### ApV1 production with protein disulfide isomerases, ER-associated flavoproteins and folding chaperones

The presence of two non-consecutive disulfide bonds in ApV1 phytase increases the chances of disulfide bonds forming between incorrect cysteine pairs. Co-expression of *ApV1* phytase with *PDI* using the BDP *P*_*HpFMD-HpMOX*_ was investigated to further improve expression. ApV1-PDI (*P*_*HpFMD-HpMOX*_) showed 14.63 ± 1.0-fold and 12.44 ± 0.09-fold increase after 132 h in phytase production compared to ApV1 (*P*_*AOX1*_) and ApV1 (*P*_*HpFMD*_) strains, respectively (Table 3). The improvement restores production to wild-type AppA production levels and strongly suggests incorrect disulfide bond formation is detrimental to ApV1 production in the absence of *PDI* over-expression. Protein disulfide isomerase homologues Mpdi and Eug1 were also tested showing a 1.41 ± 0.10-fold increase and a 0.48 ± 0.10-fold decrease in phytase production compared to ApV1 (*P*_*HpFMD*_), respectively (Fig. 4c and Table 3).

To catalyse the formation of disulfide bonds in the ER of yeast, Pdi must be restored to an oxidised state by dithiol-disulfide exchange with the membrane-associated flavoprotein Ero1. We hypothesised that the Pdi redox state could be a limiting factor for phytase disulfide bond formation. We have previously shown that Erv2 flavoprotein most likely represents an alternative pathway for disulfide bond formation, restoring the Pdi redox state and may also catalyse bond formation de novo in *P. pastoris* [38]. Ero1 and Erv2 could thereof facilitate correct folding of ApV1 phytase. ApV1-PDI-ERO1 (P_*GAP*_) and ApV1-PDI-ERV2 (P_*GAP*_) strains as well as ApV1-ERO1 (*P*_*HpFMD-HpMOX*_) and ApV1-ERV2 (*P*_*HpFMD-HpMOX*_) were constructed to study the effect of the co-expression of the flavoproteins with *ApV1* (Fig. 4d). No improvement in phytase production was observed for any of the strains (Fig. 4d and Table 3). ApV1-PDI-ERO1 (P_*GAP*_) and ApV1-PDI-ERV2 (P_*GAP*_) showed similar production of the thermostable phytase compared to ApV1-PDI (P_*HpFMD-MOX*_), suggesting the redox state of Pdi might not be limiting the correct folding of ApV1. The result suggests that either the ratio of the expressed folding catalysts (Pdi and Ero1 or Erv2) is not optimal for Pdi re-oxidation or simply that the limitation on ApV1 phytase folding is not related to the redox state of Pdi but to the isomerisation of disulfide bonds independent of Ero1 or Erv2.

Other folding catalysts not involved in disulfide bonding or restoration of the Pdi redox state were tested with *PDI* co-expression to investigate effects on ApV1 production (see Additional file 1: Table S2 and Additional file 2: Figure S1). The ER-translocation chaperone Kar2, shown previously to increase recombinant protein production [10, 20, 52, 60, 64], was expressed from the P_*GAP*_ promoter. ApV1 production decreased 0.47 ± 0.02-fold from the ApV1-PDI-KAR2 (P_*GAP*_) strain compared to the ApV1-PDI (P_*HpFMD-HpMOX*_) strain. Production of the vesicular transport proteins Sly1 and Sec1, and gluthatione peroxidase Gpx1, which has been reported to restore redox balance generated during oxidative protein folding [12, 25, 34], were also tested (see Additional file 1: Table S2 and Additional file 2: Figure S1). Production of ApV1 did not improve in any of the strains, and in fact markedly decreased, suggesting an overloading of the secretory pathway in the cell. Lastly, a strain co-expressing *PDI* and *HAC1* using the P_*CAT1*_ promoter was also tested. This ApV1-PDI-HAC1 (P_*CAT1*_) strain showed decreased ApV1 production compared to ApV1-PDI (*P*_*HpFMD-HpMOX*_) (see Additional file 1: Table S2 and Additional file 2: Figure S1).

### Influence of secretion signals on ApV1 phytase production

Three different secretion signal sequences, *Suc2*, *Pho1* and *Ost1*, were tested for ApV1 phytase production in combination with the α-factor pro region and compared to α-factor secretion signal using the ApV1-PDI (*P*_*HpFMD-HpMOX*_) strain. Fitzgerald et al., proposed that the inclusion of the α-factor pro region drives more efficient translocation of the nascent protein to the ER and facilitates rapid ER export [18, 42]. While the mechanism of translocation mediated by the *Suc2* and *Pho1* secretion signals is still not well understood, the *Ost1* secretion signal has been proposed to direct co-translational transport of nascent polypeptides into the ER of yeast [3, 18]. Due to the increased complexity of disulfide bond formation in ApV1, the use of co-translational secretion sequences might improve folding and expression compared to the post-translational α-factor signal sequence. However, the hybrid secretion sequences did not improve the expression of the phytases compared to the strain using the α-factor sequence alone (see Additional file 2: Figure S1).

### Additional disulfide bond engineering of ApV1 phytase did not further improve folding

**Fig. 5:**
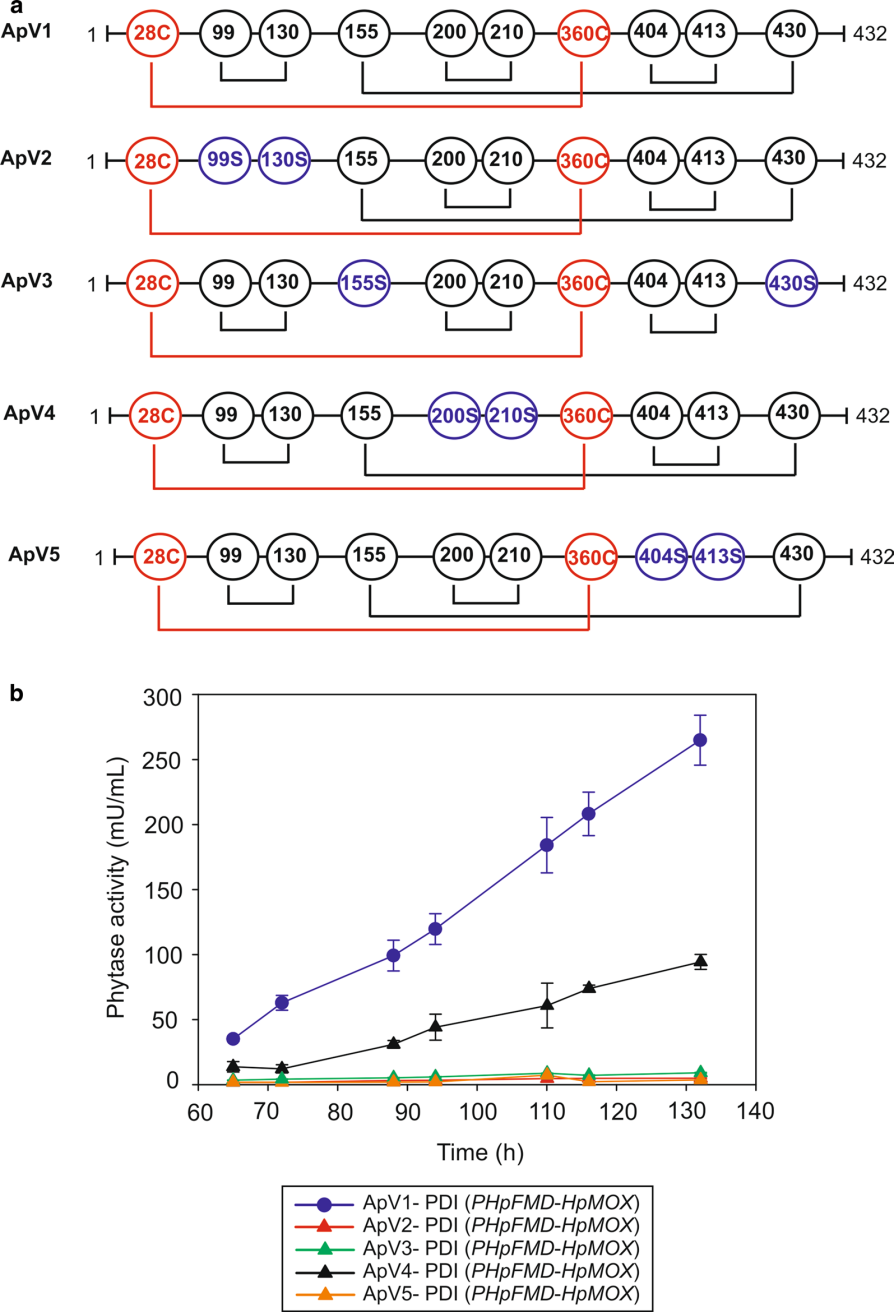
Removal of native disulfide bonds from ApV1 (variants ApV2, ApV3, ApV4 and ApV5) does not improve phytase production. Diagram showing mutated cysteine residues in each ApV1 variants (**a**). ApV2, ApV3, ApV4 and ApV5 phytase activity (mU/mL) compared to ApV1 (**b**). Phytase activity was determined by the p-NPP assay (Abs 410 nm). Data are represented as mean values ± standard deviation (*n* = 3)

Due to the difficulties in expressing the thermostable phytase due to the additional disulfide bond, further engineering of disulfide bonds was conducted in ApV1 to test for improved folding and production. Four phytase variants were constructed maintaining the ApV1 non-consecutive 28C–360C disulfide bond but eliminating one native disulfide bond in each variant by replacing each cysteine residue in the pair with a serine residue, similar to the methodology applied by Berkmen et al. [4] (Fig. 5). Variants ApV2 (C99S, C130S), ApV3 (C155S, C430S), ApV4 (C200S, C210S) and ApV5 (C404S, C413S) therefore each had a total of four disulfide bonds like the wild-type AppA. All of these phytase variants were produced with Pdi and showed decreased production in *P. pastoris* compared to AppA and ApV1 (Fig. 5b). The results suggest that the additional, non-native bond between 28 and 360C is the main reason for folding complexity and decreased production of ApV1 and that all four native disulfide bonds are important for phytase production. Berkmen et al., reported that all disulfide bonds are required for activity and that a mutation in any bond decreases phytase activity compared to the wild type. However, the elimination of the bond formed between C200 and C210 in ApV4 did not appear to decrease phytase activity (see Additional file 1: Table S3). Specific activity and kinetic parameters, as well as temperature and pH profiles, were also determined for the ApV4 phytase variant. Results showed no statistical difference in a specific activity or kinetic parameters between ApV4, ApV1 and AppA phytases (see Additional file 1: Table S3). Thermostability of ApV4 at 65, 75 and 85 °C was improved compared to AppA, however, decreased thermal stability was observed compared to ApV1 (see Additional file 3: Figure S2). The optimal temperature of activity was very similar between ApV4, ApV1 and AppA phytases (~ 50 to 55 °C) but ApV4 activity quickly decreased at temperatures above ~ 55 °C compared to ApV1 and AppA (see Additional file 3: Figure S2). Furthermore, ApV4 phytase presented lower activity at pH 2 (36 ± 3%) compared to ApV1 (80 ± 6%) and AppA (76 ± 15%) phytases and decreased pH stability after 1 h incubation at pH 2 (58 ± 3% remaining activity) (see Additional file 1: Table S4). ApV4 was more susceptible to pepsin degradation compared to ApV1 (21 ± 1% remaining activity, after 4 h incubation for ApV4 compared to 101 ± 2% remaining activity for ApV1). The accessibility to protease attack of ApV4, as well as decreased activity above ~ 55 °C, could be related to a general decrease in structural stability due to the absence of the C200–C210 disulfide bond. ApV2, ApV3 and ApV5 could not be produced in sufficient quantities to test these properties.

## Discussion

Phytases are extensively used as animal feed supplements and numerous microbial phytases are available on the market. One main challenge with phytases arises from instability and denaturation during the high-temperature steam pelleting processes that leads to loss of efficacy. In this work, we present a new thermostable variant of AppA, named ApV1. The presence of an extra disulfide bond in this phytase variant markedly decreases activity loss following incubation at elevated temperatures compared to AppA. ApV1 shows ~ 50% residual activity after 20 min following incubations up to 85 °C. Furthermore, ApV1 presents 82, 75 and 70% remaining activity at 0.5, 1 and 2 min incubation at 85 °C, respectively, while the wild-type enzyme shows 23, 17 and 11% remaining activity at the same temperature and times of incubation (Fig. 1). With feed pelleting processes involving temperatures approaching ~ 90 °C for a short period of time (up to 90 s), thermal stability during the first few seconds is crucial for enzyme survival.

The thermal stability and optimal temperature results for ApV1 suggested that the melting temperature is similar to AppA phytase. Maintaining identical activity to the wild-type enzyme at mesophilic temperatures is highly beneficial for use in livestock compared to other engineered phytases with improved stability but also increased optimal temperatures of activity and therefore decreased activity at body temperature. We hypothesise that the higher residual activity of ApV1 is associated with an increased ability to refold back to a catalytically active form after denaturation during high-temperature treatments. Quantum Blue phytase (QB) from AB Vista (identical sequence to Phy9X), is a commercially available thermostable *E. coli* AppA variant available on the market. A comparison between ApV1 with QB showed that the residual activity after thermal treatment of ApV1 is lower than that of QB at 65 and 75 °C. However, ApV1 retained similar activity to QB at 85 °C after 20 min of incubation (see Additional file 4: Figure S3).

Glycosylation contributes to the thermostability of enzymes [23, 27, 58] and may prevent the unfolded or partially folded protein molecules from aggregation during heat treatment [47, 48]*.* Thermostability curves of deglycosylated ApV1 phytase showed decreased residual activity compared to the glycosylated counterpart. However, deglycosylated ApV1 retained improved thermal stability compared to AppA supporting the idea of an intrinsic mechanism related to the extra disulfide bond in ApV1 enhancing refolding that is independent of glycan chains.

Common strategies to increase thermal stability based on the incorporation of several mutations in the protein sequences [11, 30, 63] can potentially result in changes in catalytic properties that are not desirable for animal feed applications, including a decrease in activity at mesophilic temperatures. Detailed biochemical characterisation of ApV1 showed no statistical activity difference to AppA phytase, confirming that desirable properties such as optimal temperature of activity, catalytic efficiency, *K*_M_ and pH stability of the phytase are not affected by the incorporation of the extra disulfide bond.

While the incorporation of the extra disulfide bond in ApV1 greatly increased thermal stability it also had a very strong negative effect on production in *P. pastoris*. Decreased production was most likely associated with the mispairing of cysteines during the formation of the disulfide bonds and the redox cost of extra isomerisation steps required to correct cysteine mispairing. During the formation of native bonds, disulfide isomerisation occurs by breakage of incorrectly formed bonds and creation of new cysteine pairs until the right combination is found. Mispairing of cysteines can lead to inactive enzyme and aggregation which subsequently triggers the UPR and rapid degradation by ER-associated degradation (ERAD) [2]. Western Blot studies did not show accumulation of intracellular aggregated ApV1 phytase (data not shown) suggesting a rapid degradation of misfolded protein (if present at all) by the cellular machinery.

A synergistic approach for transcriptional expression and protein folding optimisation was conducted using methanol inducible and de-repressed MDPs as well as BDPs for co-expression of chaperones and folding catalysts. The co-expression of ApV1 with disulfide bond isomerases, ER-membrane flavoproteins, folding chaperones, vesicular transport proteins and ROS detoxification was directed to address potential bottlenecks for ApV1 production in *P. pastoris*.

Co-expression of *PDI* using *P*_*HpFMD-HpMOX*_ showed improved phytase yields. Production was ~ 13-fold increased using ApV1-PDI (*P*_*HpFMD-HpMOX*_) compared to ApV1 (*P*_*HpFMD*_) suggesting that over-expression of the isomerase is essential for breaking and re-forming disulfide bonds to generate the active phytase (Fig. 4). In line with this observation, the production of Pdi homologues (Mpdi and Eug1) did not improve ApV1 production [41].

We previously reported that co-expression of *ERV2* or *ERO1* flavoproteins increased the production of AppA phytase, most likely due to restoration of the Pdi redox state [38]. In the case of ApV1, no improvement was observed when the flavoproteins were produced with Pdi. The limiting factor in ApV1 production may therefore be related to the extra disulfide bond and the associated additional isomerisation steps required for correct folding and activity rather than being directly related to restoration of the Pdi redox state required for de novo formation of disulfide bonds.

Apart from the isomerisation of disulfide bonds, the co-expression of *PDI* has been associated with changes in glycosylation patterns [40]. We considered that the production of Pdi in ApV1-PDI (*P*_*HpFMD-HpMOX*_) could influence phytase glycosylation resulting in further improved thermostability of ApV1 as well as improved folding. The thermostability of ApV1 phytase produced during co-expression with *PDI* was compared to ApV1 phytase produced without chaperone co-expression from ApV1 (*P*_*HpFMD*_) strain. Notably, co-expression of *PDI* showed improved thermostability of the phytase compared to no chaperone co-expression. ApV1 phytase showed ~ 50% residual activity after 20 min at 65, 75 and 85 °C (Fig. 2), while 20 to 30% remaining activity was observed when the phytase was produced without Pdi under the same conditions (see Additional file 5: Figure S4). The thermostability increase observed for ApV1-PDI strain might indicate a difference in glycosylation during folding of phytase, however, no changes in electrophoretic mobility were detected between phytases produced with or without Pdi (see Additional file 7: Figure S6).

To further improve production of the thermostable phytase, chaperones associated with folding, trafficking and redox homeostasis were also co-produced with ApV1 showing no improvement in production (see Additional file 6: Figure S5). Chaperone Kar2 binds to the nascent polypeptide helping in folding and translocation to the ER. Since the folding process is based on trial and error until the correct conformation is found, proteins bound to Kar2 for too long can trigger ERAD [43]. This mechanism could explain the decreased production of ApV1 in ApV1-PDI-KAR2 (P_*GAP*_) strain.

Further disulfide bond engineering in ApV1 was conducted by maintaining the new disulfide bond whilst individually removing each of the native disulfide bonds. This experiment aimed to assess whether improved thermostability could be maintained with the second non-sequential disulfide bond (28C–360C) in a protein that had the same overall number of disulfide bonds as the wild-type AppA. All new variants, however, showed decreased expression (Fig. 5) and phytase activity was reduced in the new variants except for the ApV4 phytase, showing that all native disulfide bonds are important for expression and/or activity in line with previous reports [4]. Biochemical characterisation of *K*_M_ and catalytic efficiency of the ApV4 variant showed no statistical difference with ApV1 or AppA phytases (see Additional file 1: Table S3), however, the pH and temperature profile, thermostability and pepsin resistance were negatively affected. The result suggests that the C200–C210 disulfide bond is important for protein stability and contributes to refolding after heat stress, and that the extra 28C–360C disulfide bond is not sufficient to compensate for the loss of C200–C210.

## Conclusions

The work presented shows detailed characterisation of the ApV1 thermostable variant of *E. coli* AppA phytase that incorporated an additional non-consecutive disulfide bond. The variant is an excellent candidate for animal feed applications due to increased activity following exposure to temperatures experienced during feed pellet manufacture using steam while maintaining the same enzymatic properties at livestock gut temperatures. The presence of the extra non-consecutive disulfide bond strongly hinders production in *P. pastoris* but this was alleviated by co-expression of *PDI* using a bi-directional promoter (*P*_*HpFMD-HpMOX*_).

This study shows that enhancement of enzymatic properties, like incorporation of additional disulfide bonds for improved thermal stability, may result in folding complexity and so parallel rational strain engineering is required to achieve desirable levels of production of such improved enzymes to meet industrial production requirements*.*

## Methods

### Strains and growth conditions

The strain used in this study was the *P. pastoris* BG11 strain (derivative of *P. pastoris* BG10 strain, *ΔAOX1* (mutS—methanol utilisation slow) from ATUM Inc. (Newark, California, USA). α-Select Silver efficiency competent *E. coli* strain (Bioline, Australia) was used for cloning. Cultivations were conducted in Luria Broth (LB) media for *E. coli* and yeast cultures were either grown in YPD medium (1% w/v yeast extract, 2% w/v peptone and 2% w/v glucose), buffered minimal dextrose (BMD) medium (1.34% Yeast Nitrogen Base YNB, 4 × 10^−5^% biotin, 200 mM potassium phosphate buffer pH 6.0 and 2% glucose), buffered minimal methanol (BMM) medium (1.34% YNB, 4 × 10^−5^% biotin, 200 mM potassium phosphate buffer pH 6.0) with 1% methanol (BMM2) or 5% methanol (BMM10). Antibiotic Zeocin (Invitrogen) was added to the media when required at a final concentration of 25 µg/mL for *E. coli* or 100 µg/mL for *P. pastoris* cultivations. Small scale cultivations of *P. pastoris* were performed using deep-well-plates (DWP) and induction protocols reported previously [59].

### Computational modelling of ApV1 phytase

Molecular modelling was performed in a previous collaboration between Bioproton Pty Ltd., The University of Queensland, Brisbane and Sanoosa Pty Ltd (formerly Qubist Molecular Design Pty Ltd). Existing structural data from fungal and microbial phytases were used to assess the basis for heat stability and to guide rational design modifications to the *E. coli* AppA wild type phytase and generate the ApV1 phytase (data not published).

### Cloning and transformation of *Pichia pastoris*

The vector used for cloning and integration into *P. pastoris* genome was pD912 from ATUM Inc., which is based on the pPpT4S vector family [37]. All plasmids containing monodirectional promoter (MDP) sequences *P*_*AOX1*_, the orthologous formate dehydrogenase promoter from *Hansenula polymorpha* (*P*_*HpFMD*_) [56], BDP sequences *P*_*DAS1/2*_, *P*_*HpFMD-HpMOX*_ and *P*_*AOX1-CAT1*_ [57], chaperone genes *HAC1* (spliced version) [22], *PDI* (accession number ACF17572.1), *ERV2* (accession number ANZ73509.1), *MPDI* (accession number XP_002489466), *ERO1* (accession number ANZ74048.1), *KAR2* (accession number ANZ77450.1), *GPX1* (accession number BAH57503.1), *SEC1* (accession number CAY67514.1), *SLY1* (accession number CCA40908.1), and *EUG1* from *S. cerevisiae* (accession number XP_446874) were derived from vectors described in Navone et al. [38]. The *appA* wild-type gene sequence was replaced by the gene sequence encoding ApV1 by Gibson assembly where indicated. The gene encoding the ApV1 phytase was codon optimised by ATUM Inc. and expressed in the direction of the first promoter region indicated in the BDP name. Chaperones were expressed in the direction of the second promoter region indicated in the BDP name (see Additional file 1: Table S1). The α-mating factor secretion signal from *S. cerevisiae* was used unless indicated [7]. Secretion signals *Suc2*, *Pho1*, *Ost1* included the α-factor pro-region at the 3′ end. Genes encoding the ApV2, ApV3, ApV4 and ApV5 phytase variants were ordered as gBlocks from Integrated DNA Technologies (IDT), Australia, with 50 bp overlapping sequences at the 5′ and 3′ termini for Gibson assembly with pD912 derived plasmids. *P. pastoris* cells were transformed with SwaI linearised plasmids following a condensed standard protocol [33] with 1–3 µg of total DNA. After the transformation, screening of transformants was performed in deep-well plates as described previously [59].

### Phytase production and purification

ApV1, AppA and ApV4 phytase productions were conducted in 250 mL baffled shake flasks following standard expression conditions at 28 °C, 250 rpm. The cultures were grown using 50 mL of BMD1 for 65 h following methanol induction using BMM10 and consecutive additions of pure methanol 1% final concentration until harvest at 132 h. The supernatant was loaded onto a Ni-Sepharose ion affinity chromatography HisTrap FF columns (GE Healthcare) for phytase purification. Binding and elution were done following the manufacturer’s instructions and purified phytase was buffer exchanged in 250 mM sodium acetate pH 5.5 for kinetic determinations using HiTrap Desalting column (GE Healthcare) following the manufacturer’s instructions. Protein concentrations were determined using the Bradford method [5]. For deglycosylation of purified phytases, 100 μg of total protein was incubated with 0.5 units of endoglycosidase Hf (Endo Hf) for 4 h at 37 °C according to the manufacturer’s instructions (New England Biolabs, Australia).

### Genomic DNA extraction and gene copy number determination

Genomic DNA was extracted using the ISOLATE II Genomic DNA kit (Bioline Pty Ltd.; Alexandria, Australia) and gene copy number determination was performed as described previously [6] using Bio-Rad QX200 Droplet Digital PCR (ddPCR). Briefly, genomic DNA was digested with the SphI restriction enzyme (New England Biolabs) and 0.5 ng of digested DNA of each sample was added to the reaction mixture. For DNA amplification of the *APV1* phytase gene a set of primers (Fwd 5′-TTGTCCTCAATCCGGTCAAG-3′ and Rev 5′-AGGGTTAAACAACGGATCGG-3′) and a 5′ hexachloro-fluorescein (HEX) dye-labelled probe were designed (5′-HEX-TTGGCTCCAGACTGTGCTATCACTGT-IB®FQ-3′). The arginosuccinate lyase (*ARG4*) gene was used as reference gene and a set of primers (Fwd 5′-TGCGGTTGTATGTCAGAGAC-3′ and Rev 5′-GGTTGAGCTCTTTGCAAGTG-3′) and a 5′ 6-fluorescein amidite (6-FAM) dye-labelled probe were designed (5′-FAM-TGGCTGACTATCTGAAGCAGTTCATTCA-IB®FQ-3′) [1]. The probes were also labelled with Iowa Black® FQ at the 3′ terminus. All primers and probes were synthesised by IDT. Each PCR was performed in a 20 µL volume containing 12.5 µL of ddPCR Supermix for Probes (Bio-rad), 900 nM of each primer, 250 nM of each probe, 0.5 ng of digested genomic DNA and the required amount of MilliQ water. Thermal cycle conditions were 95 °C (10 min), 95 °C (30 s) and 58 °C (1 min) for 40 cycles, then 98 °C (10 min). Droplet detection was carried out using QuantaSoft software (Bio-Rad) and the gene copy number was calculated for each sample in triplicate.

### Enzyme activity determination

#### para-Nitrophenyl phosphate assay

Acid phosphatase activity was assayed using *para*-nitrophenyl phosphate (p-NPP) (Sigma) at a concentration of 5 mM [15]. Briefly, 10 µL of five enzyme dilutions were incubated with 90 µL of p-NPP substrate in 250 mM sodium acetate buffer pH 5.5 for 15 min at 37 °C. The reaction was stopped with the addition of 10 µL of 1 M NaOH and incubation at room temperature for 10 min. The released *para*-nitrophenol was measured at 410 nm. Reactions were conducted in triplicate in 96-well plates. The initial concentrations of p-NPP used for the enzyme kinetics were 0.15, 0.3, 0.6, 1.25, 2.5, 5, 10 and 20 mM. One unit of acid phosphatase activity was defined as the amount of enzyme catalysing the formation of 1 µmol of *para*-nitrophenol per minute.

#### Molybdate assay

Phytase activity was measured using sodium phytate (Sigma-Aldrich) as the substrate following the protocol previously described [26]. Briefly, 40 µL of 5 enzyme dilutions were incubated with 80 µL of 12 mM sodium phytate in 250 mM sodium acetate buffer pH 5.5 for 30 min at 37 °C. The reaction was stopped by the addition of 80 µL of stop reagent containing ammonium vanadate (0.118 g in 50 mL) and ammonium heptamolybdate (5 g in 50 mL) in nitric acid. The phosphate released from phytate was determined by the formation of a yellow complex with an acidic molybdate-vanadate reagent. The optical density of the yellow complex was measured at 415 nm and the inorganic phosphate released quantified using a phosphate standard curve.

One phytase unit was defined as the amount of activity that releases 1 µmol of phosphate from sodium phytate per minute at 37 °C. Reactions were conducted by triplicate in 96-well-plates. The initial concentrations of sodium phytate used for the enzyme kinetic studies were 0.2, 0.4, 0.75, 1.5, 3, 6, 10, and 12 mM.

#### Temperature and pH profiles

For temperature activity profiles, phytase reactions were incubated in 96-well-plates with p-NPP as described for the p-NPP assay at temperatures between 30 and 90 °C using a gradient setting in a PCR thermocycler (Eppendorf™ Mastercycler™ pro PCR System). For pH activity profiles, phytase reactions were performed in 96-well-plates as described for the p-NPP assay at varying pH values (Glycine–HCl buffer 250 mM pH 1, 2, 3 and 3.5, sodium acetate buffer 250 mM pH 4, 4.5, 5, 5.5 and 6, Tris-Base buffer 250 mM pH 7, 8, 9 and 10) for 15 min at 37 °C. Relative activity was calculated as the percentage of phytase activity compared to the optimal temperature or pH.

#### Thermostability assay

For the thermostability assay, the enzyme was incubated for 0.5, 1, 2, 4, 8, 12 and 20 min at 65, 75 and 85 °C. The samples were cooled on ice immediately after incubation for 30 min before conducting the p-NPP assay to determine residual activity. Residual activity was calculated as a percentage of the initial activity without heat incubation.

#### pH stability and pepsin resistance assay

For pH stability studies, enzymes were incubated in 0.25 M glycine–HCl buffer pH 1, 2, 3 or 3.5 or 0.25 M sodium acetate buffer pH 4, 4.5, or 5 for 1 h at 37 °C. For protease resistance determinations, phytase samples were incubated with pepsin (Sigma-Aldrich) in a 1/1 mass ratio of phytase/pepsin at 37 °C for 4 h. Phytase activity was determined immediately after incubations by the p-NPP assay. Remaining activity was calculated as a percentage of the phytase activity at optimal pH incubation or without pepsin treatment.

## Supplementary Information

**Additional file 1: Table S1.** Plasmid design and promoters. **Table S2.** Fold change of ApV1 phytase production relative to *P*_*AOX1*_, P_*HpFMD*_ and P_*HpFMD-HpMOX*_. **Table S3.** Kinetic parameters for ApV4 phytase. **Table S4.** pH stability of ApV4 phytase.

**Additional file 2: Figure S1.** α-factor secretion signal shows the highest yields in ApV1 phytase.

**Additional file 3: Figure S2.** ApV4 showed decreased thermostability and different pH and temperature profiles to AppA and ApV1 phytases.

**Additional file 4: Figure S3.** ApV1 and QB phytases thermostability curves.

**Additional file 5: Figure S4.** ApV1 thermostability improves with co-expression of PDI.

**Additional file 6: Figure S5.** *ApV1* co-expression with *PDI* and *KAR2*, *GPX1*, *SEC1*, *SLY1* or *HAC1* does not improve production.

**Additional file 7: Figure S6.** SDS-PAGE of ApV1 and AppA phytases.

## Data Availability

The datasets used and/or analysed in this study are included in this published article and its Additional files.
