# Supplementary material for: Disulfide bond engineering of AppA phytase for increased thermostability requires co-expression of protein disulfide isomerase in Pichia pastoris
Source: Biotechnol Biofuels. 2021 Mar 31;14:80. doi: 10.1186/s13068-021-01936-8 (PMC8010977; doi:10.1186/s13068-021-01936-8)
Supplement: Supplementary file 1 — Additional file 1: Table S1. Plasmid design and promoters. Table S2. Fold change of ApV1 phytase production relative to PAOX1, PHpFMD and PHpFMD-HpMOX. Table S3. Kinetic parameters for ApV4 phytase. Table S4. pH stability of ApV4 phytase. [file 13068_2021_1936_MOESM1_ESM.docx]

**Table S1. Plasmid design and promoters.**

| **Strain name** | **Phytase promoter** | **Phytase** | **Chaperone 1 promoter** | **Chaperone 1** | **Chaperone 2 promoter** | **Chaperone 2** | **Selection marker** |
| --- | --- | --- | --- | --- | --- | --- | --- |
| AppA (P_AOX1_) | P_AOX1_ | ApV1 | - | - | - | - | Zeocin |
| ApV1 (P_HpFMD_) | P_HpFMD_ | ApV1 | - | - | - | - | Zeocin |
| ApV1-HAC1 (P_HpFMD-HpMOX_) | P_HpFMD_ | ApV1 | P_HpMOX_ | HAC1 | - | - | Zeocin |
| ApV1-HAC1 (P_AOX1-CAT1_) | P_AOX1_ | ApV1 | P_CAT1_ | HAC1 | - | - | Zeocin |
| ApV1-HAC1 (P_DAS1/2_) | P_DAS1_ | ApV1 | P_DAS2_ | HAC1 | - | - | Zeocin |
| ApV1-PDI (P_HpFMD-HpMOX_) | P_HpFMD_ | ApV1 | P_HpMOX_ | PDI | - | - | Zeocin |
| ApV1-MPDI (P_HpFMD-HpMOX_) | P_HpFMD_ | ApV1 | P_HpMOX_ | MPDI | - | - | Zeocin |
| ApV1- EUG1 (P_HpFMD-HpMOX_) | P_HpFMD_ | ApV1 | P_HpMOX_ | EUG1 | - | - | Zeocin |
| ApV1-SEC1 (P_HpFMD-HpMOX_) | P_HpFMD_ | ApV1 | P_HpMOX_ | SEC1 | - | - | Zeocin |
| ApV1-SLY1 (P_HpFMD-HpMOX_) | P_HpFMD_ | ApV1 | P_HpMOX_ | SLY1 | - | - | Zeocin |
| ApV1-ERV2 (P_HpFMD-HpMOX_) | P_HpFMD_ | ApV1 | P_HpMOX_ | ERV2 | - | - | Zeocin |
| ApV1-ERO1 (P_HpFMD-HpMOX_) | P_HpFMD_ | ApV1 | P_HpMOX_ | ERO1 | - | - | Zeocin |
| ApV1-PDI-ERO1 (P_GAP_) | P_HpFMD_ | ApV1 | P_HpMOX_ | PDI | P_GAP_ | ERO1 | Zeocin |
| ApV1-PDI-ERV2 (P_GAP_) | P_HpFMD_ | ApV1 | P_HpMOX_ | PDI | P_GAP_ | ERV2 | Zeocin |
| ApV1-PDI- GPX1 (P_GAP_) | P_HpFMD_ | ApV1 | P_HpMOX_ | PDI | P_GAP_ | GPX1 | Zeocin |
| ApV1-PDI- HAC1 (P_CAT1_) | P_HpFMD_ | ApV1 | P_HpMOX_ | PDI | P_CAT1_ | HAC1 | Zeocin |
| ApV1-PDI- KAR2 (P_GAP_) | P_HpFMD_ | ApV1 | P_HpMOX_ | PDI | P_GAP_ | KAR2 | Zeocin |

**Table S2. Fold change of ApV1 phytase production relative to *P_AOX1_*, P*_HpFMD_* and P*_HpFMD-HpMOX_*.**

| **Strain** | **Fold change relative to ApV1 (*P_AOX1_*).** | **Fold change relative to ApV1 (*P_HpFMD_*).** | **Fold change relative to ApV1-PDI (*P_HpFMD-MOX_*).** |
| --- | --- | --- | --- |
| APV1-PDI-GPX1 (P*_GAP_*) | 15.57 ± 1.05* | 13.23 ± 0.10** | 1.07 ± 0.07 |
| APV1-PDI-KAR2 (P*_GAP_*) | 6.81 ± 0.30* | 4.23 ± 0.25** | 0.47 ± 0.02*** |
| APV1-PDI-HAC1 (P*_CAT1_*) | 5.61 ± 0.50* | 4.77 ± 0.43** | 0.38± 0.03*** |
| APV1-PDI-SLY1 (P*_CAT1_*) | 3.82 ± 0.45* | 3.25 ± 0.38** | 0.26 ± 0.03** |
| APV1-PDI-SEC1 (P*_CAT1_*) | 3.31 ± 0.18* | 2.82 ± 0.15** | 0.23 ± 0.17*** |

**Table S3. Kinetic parameters for ApV4 phytase.**

| **Molybdate assay** | | | | |
| --- | --- | --- | --- | --- |
|  | **Specific activity (10^3^ U/g of protein)*** | **Km (mM)*** | **kcat (min^-1^)*** | **kcat/km (min^-1^M^-1^)*** |
| ApV4 phytase | 1243 ± 165 | 0.63 ± 0.19 | 2.20 x 10^5^ ± 2.77 x 10^4^ | 1.81 x 10^8^± 9.73 x 10^7^ |
| **p-NPP assay** | | | | |
| ApV4 phytase | 998 ± 23 | 14.04 ± 0.50 | 1.84 x 10^5^ ± 6.50 x 10^4^ | 1.30 x 10^8^± 4.17 x 10^6^ |

*No significant statistical difference between ApV4 and AppA phytase for molybdate or p-NPP assays (p ≤ 0.05). **Values correspond to biological triplicates of his-tag purified ApV4 phytase.

**Table S4. pH stability of ApV4 phytase**

|  | **pH 2** | **pH 3** | **pH 4** | **pH 5** |
| --- | --- | --- | --- | --- |
| ApV4 | 58 ± 3 | 105 ± 9 | 97 ± 4 | 104 ± 3 |
