# Supplementary material for: Disulfide bond engineering of AppA phytase for increased thermostability requires co-expression of protein disulfide isomerase in Pichia pastoris
Source: Biotechnol Biofuels. 2021 Mar 31;14:80. doi: 10.1186/s13068-021-01936-8 (PMC8010977; doi:10.1186/s13068-021-01936-8)
Supplement: Supplementary file 2 — Additional file 2: Figure S1. α-factor secretion signal shows the highest yields in ApV1 phytase. [file 13068_2021_1936_MOESM2_ESM.docx]

**
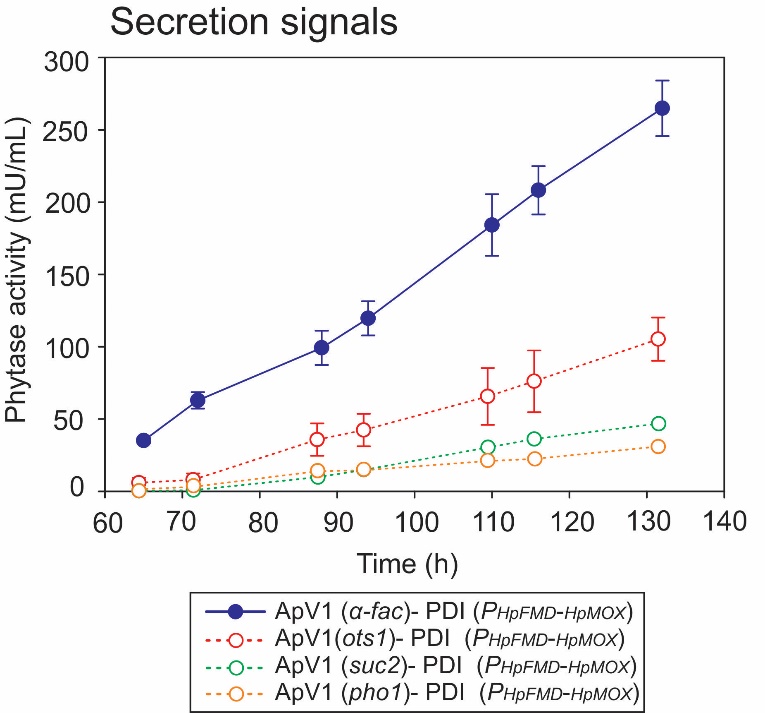
**

**Fig S1. α- factor secretion signal shows the highest yields in ApV1 phytase.** ApV1 phytase production with α-factor, ApV1 (*α-fac*)-PDI (*P_HpFMD-HpMOX_*), *suc2*, ApV1 (*suc2*)-PDI (*P_HpFMD-HpMOX_*), *ost1,* ApV1 (*ost1*)-PDI (*P_HpFMD-HpMOX_*) or *pho1,* ApV1 (*pho1*)-PDI (*P_HpFMD-HpMOX_*) secretion signals. Phytase activity was determined by the p-NPP assay (Abs 410 nm). Data are represented as mean values ± standard deviation (n=3).
