# Supplementary material for: Disulfide bond engineering of AppA phytase for increased thermostability requires co-expression of protein disulfide isomerase in Pichia pastoris
Source: Biotechnol Biofuels. 2021 Mar 31;14:80. doi: 10.1186/s13068-021-01936-8 (PMC8010977; doi:10.1186/s13068-021-01936-8)
Supplement: Supplementary file 3 — Additional file 3: Figure S2. ApV4 showed decreased thermostability and different pH and temperature profiles to AppA and ApV1 phytases. [file 13068_2021_1936_MOESM3_ESM.docx]

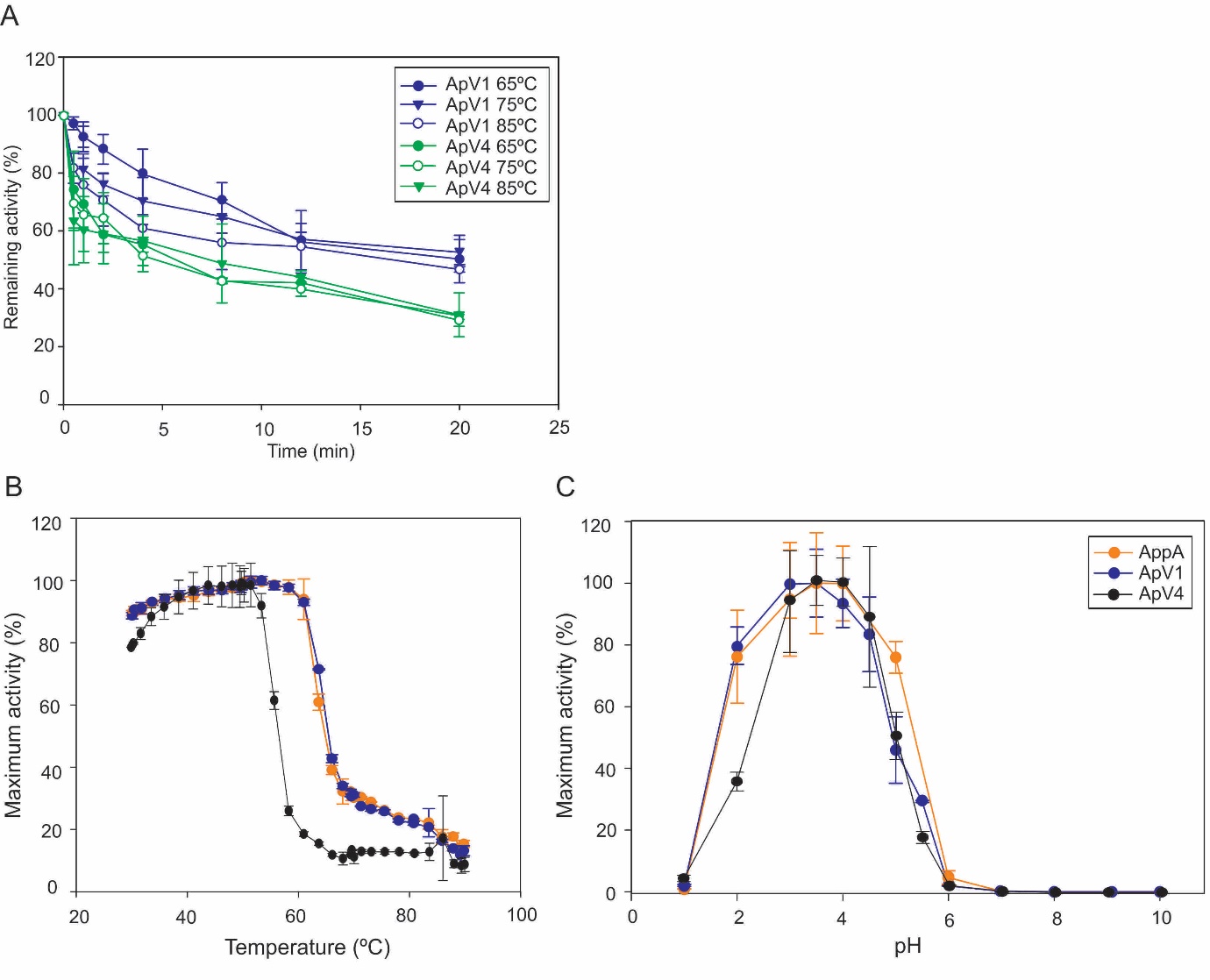


**Fig. S2. ApV4 showed decreased thermostability and different pH and temperature profiles to AppA and ApV1 phytases.** Remaining activity of ApV1 and ApV1 phytases (A), optimal temperature (A) and pH (B) curves for ApV4, ApV1 and AppA phytases. Phytase activity was determined by the p-NPP assay after incubation at 65, 75 or 85⁰C. Remaining activity was calculated as a percentage of phytase activity without high temperature treatment. Percentage of maximum activity was calculated as a percentage of phytase activity at the optimal temperature or pH. Data are represented as mean values ± standard deviation (n=3).
