# Supplementary material for: Disulfide bond engineering of AppA phytase for increased thermostability requires co-expression of protein disulfide isomerase in Pichia pastoris
Source: Biotechnol Biofuels. 2021 Mar 31;14:80. doi: 10.1186/s13068-021-01936-8 (PMC8010977; doi:10.1186/s13068-021-01936-8)
Supplement: Supplementary file 4 — Additional file 4: Figure S3. ApV1 and QB phytases thermostability curves. [file 13068_2021_1936_MOESM4_ESM.docx]

**
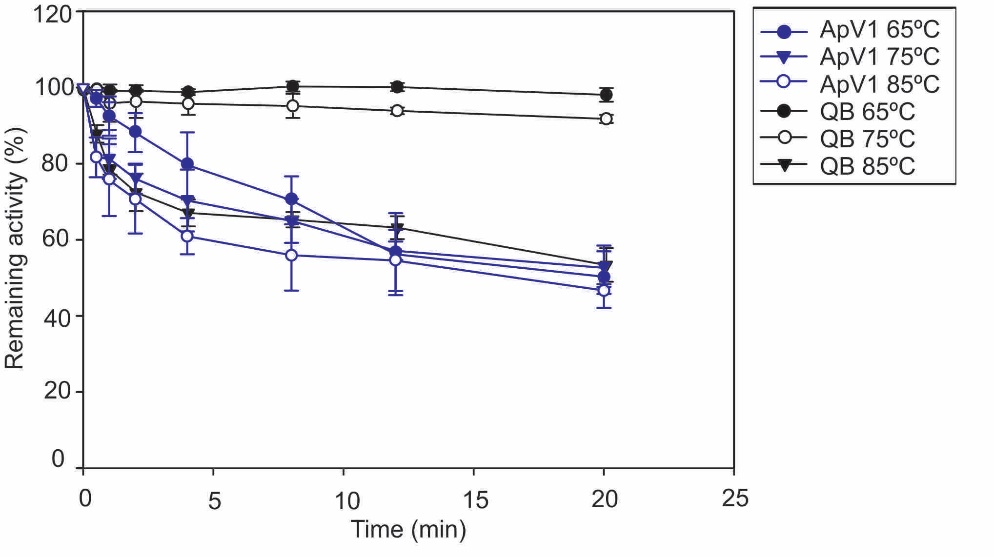
**

**Fig S3. ApV1 and QB phytases thermostability curves.** Remaining activity of ApV1 and QB phytases. Phytase activity was determined by the p-NPP assay after incubation at 65, 75 or 85⁰C. Remaining activity was calculated as a percentage of phytase activity without high temperature treatment. Data are represented as mean values ± standard deviation (n=3).
