# Supplementary material for: Disulfide bond engineering of AppA phytase for increased thermostability requires co-expression of protein disulfide isomerase in Pichia pastoris
Source: Biotechnol Biofuels. 2021 Mar 31;14:80. doi: 10.1186/s13068-021-01936-8 (PMC8010977; doi:10.1186/s13068-021-01936-8)
Supplement: Supplementary file 5 — Additional file 5: Figure S4. ApV1 thermostability improves with co-expression of PDI. [file 13068_2021_1936_MOESM5_ESM.docx]

*
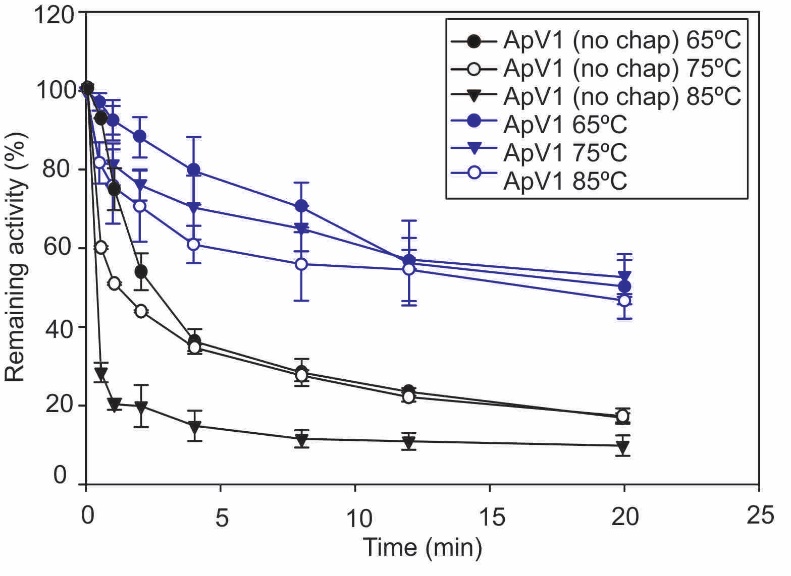
*

**Fig S4. ApV1 thermostability improves with co-expression of PDI.** Phytase activity was determined by the p-NPP assay after incubation at 65, 75 or 85⁰C. ApV1 (no chap) indicates ApV1 expressed without chaperone. Remaining activity was calculated as a percentage of phytase activity without high temperature treatment. Data are represented as mean values ± standard deviation (n=3).

after methanol induction. Data are represented as mean values ± standard deviation (n=3).
