# Supplementary material for: Disulfide bond engineering of AppA phytase for increased thermostability requires co-expression of protein disulfide isomerase in Pichia pastoris
Source: Biotechnol Biofuels. 2021 Mar 31;14:80. doi: 10.1186/s13068-021-01936-8 (PMC8010977; doi:10.1186/s13068-021-01936-8)
Supplement: Supplementary file 6 — Additional file 6: Figure S5. ApV1 co-expression with PDI and KAR2, GPX1, SEC1, SLY1 or HAC1 does not improve production. [file 13068_2021_1936_MOESM6_ESM.docx]

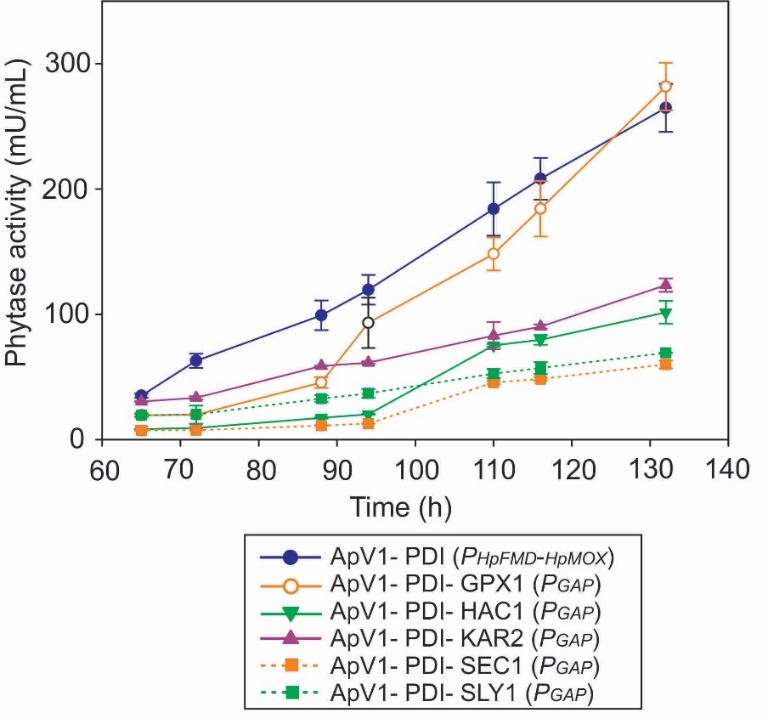


**Fig. S5. ApV1 co-expression with PDI and KAR2, GPX1, SEC1, SLY1 or HAC1 does not improve production.** Phytase expression determined by the p-NPP assay after methanol induction. Data are represented as mean values ± standard deviation (n=3).
