# Supplementary material for: Disulfide bond engineering of AppA phytase for increased thermostability requires co-expression of protein disulfide isomerase in Pichia pastoris
Source: Biotechnol Biofuels. 2021 Mar 31;14:80. doi: 10.1186/s13068-021-01936-8 (PMC8010977; doi:10.1186/s13068-021-01936-8)
Supplement: Supplementary file 7 — Additional file 7: Figure S6. SDS-PAGE of ApV1 and AppA phytases. [file 13068_2021_1936_MOESM7_ESM.docx]

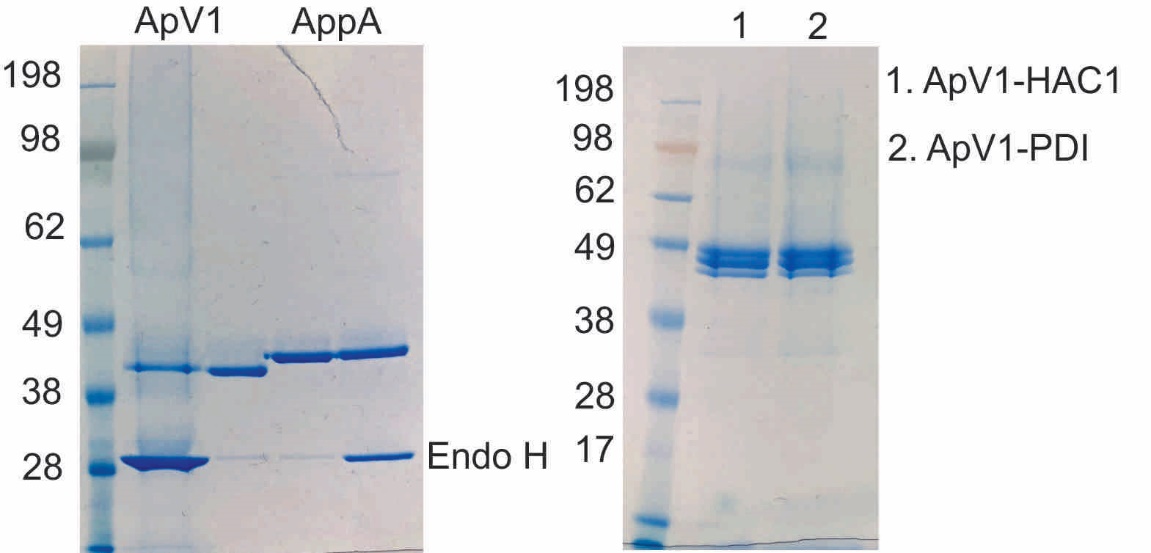


**Fig. S6. SDS-PAGE of ApV1 and AppA phytases.** SDS-PAGE under non-reducing conditions of deglycosylated ApV1 and AppA phytases. The presence of the extra disulfide bond in ApV1 changes electrophoretic mobility under non-reducing conditions (SDS-PAGE on the left). Glycosylation patterns of ApV1 phytase produced with Hac1 or Pdi. No difference in glycosylation patterns were observed between the samples (SDS-PAGE on the right).
